# Advances in Forest Ecophysiology: Stress Response and Ecophysiological Indicators of Tree Vitality

**DOI:** 10.3390/plants12051063

**Published:** 2023-02-27

**Authors:** Nenad Potočić

**Affiliations:** Croatian Forest Research Institute, Division of Forest Ecology, Cvjetno naselje 41, 10450 Jastrebarsko, Croatia; nenadp@sumins.hr

Back in the beginning of the year 2021, when the work on this Special Issue started, it was quite clear that the topics of tree stress response and the ecophysiological indicators of tree vitality were both current and important, but the attitude of the scientific community towards the idea of a Special Issue on the subject was yet to be determined. Now that we are closing the first, and already preparing the second volume, I am very happy to definitely confirm your high interest.

Air pollution and the changing climate are some of the greatest threats to the health and functioning of forest ecosystems, strongly jeopardizing their ecological and economic functions as well as services. The impact of increasing temperatures and extreme weather events (droughts, storms, temperature and precipitation extremes) on the vitality of forest trees is often difficult to separate from the impact of pollution, such as nitrogen deposition and tropospheric ozone, as they can exhibit synergistic effects. For example, forest soil acidification, atmospheric N deposition, and climate change are all partly responsible for the continuous decrease in foliar P concentrations in Europe, causing reduced tree growth [1,2]. The use of indicators is elementary in modern forest ecophysiological research, as they help us to disentangle complex interactions between trees and various stress-inducing factors as well as better estimate the level of damage to trees and forest ecosystems.

The initial Special Issue framework, as defined by the title and suggested topics of interest, has been broadened in the process thanks to your valuable submissions, adding terms such as heavy metals, carbon isotopes, water use efficiency, polyamines, antioxidants, or plant hormones to the list of subjects which already included photosynthetic activity and other biochemical stress indicators; nutrients in different tree compartments; tree growth; tree leaf loss and mortality; visible symptoms of stress in foliage; and microscopical markers of stress. All of these terms share one common trait: they have an important role in measuring and assessing tree stress in the context of the great ecological challenges of today. 

Eleven papers are included in this Special Issue, with wide-ranging topics from various disciplines but centered around tree response to environmental stress. In line with the current trends in environmental research, climate takes clear precedence over pollution, and as many as nine of the articles discuss climate effects; tree anatomy, growth, nutrition, foliar injury, the level of antioxidants, and defoliation are used as response variables. 

Three papers, by Levanič and Štraus [3], Sensuła and Wilczyński [4], and Popa et al. [5], discuss the effects of climate on tree growth, and the paper by Sensuła and Wilczyński [4] also includes the effects on water use efficiency and carbon isotopes’ composition. Two papers, one by Ognjenović et al. [6] and another by Češljar et al. [7], focus on climate effects on tree crown defoliation. While the first paper tests the differences between defoliation and defoliation change as long-term and short-term indicators, respectively, of tree vitality, the second paper discusses the links between crown defoliation and tree mortality. Defoliation is again featured in the paper by Ognjenović et al. [8], where its effect on the nutritional response of beech trees to climate and stand factors is discussed. Soheili et al. [9] investigate how cell anatomical structure is affected by drought stress. Nutrition, oxidative stress, and defoliation in four Mediterranean tree species are the focus of the paper by Lovreškov et al. [10], while Kebert et al. [11] discuss species-level differences in osmoprotectants and antioxidants under water deficit and high temperatures. Despite the prevalence of interest in climate effects, pollution-oriented papers are not less exciting: tree responses to heavy-metal-induced stress are the subject of a paper by Kebert et al. [12], and the effects of ozone and drought on oak seedlings are discussed by Baesso Moura et al. [13]. The latter paper also nicely underlines the fact that climate and pollution effects on trees, although often investigated separately, are in practice often related and intertwined. 

In conclusion, the task of this Special Issue is twofold: one, to remind us that a better understanding of the physiological processes influencing tree vitality under the changing climate and air pollution pressures requires considerable research efforts and constant advancements in research methods and approaches; two, to highlight the fact that the environmental pressures instigating the use of tree stress response indicators are more present than ever, and will likely continue to affect tree vitality in the foreseeable future.

As the Guest Editor of this Special Issue, I would like to use the opportunity to thank all of the authors for their valuable submissions, their dedicated and hard work, and the continuous exchange of thoughts and ideas that form the backbone of current scientific research, pushing it ever further.
